# Patient Characteristics Associated With Telemedicine Access for Primary and Specialty Ambulatory Care During the COVID-19 Pandemic

**DOI:** 10.1001/jamanetworkopen.2020.31640

**Published:** 2020-12-29

**Authors:** Lauren A. Eberly, Michael J. Kallan, Howard M. Julien, Norrisa Haynes, Sameed Ahmed M. Khatana, Ashwin S. Nathan, Christopher Snider, Neel P. Chokshi, Nwamaka D. Eneanya, Samuel U. Takvorian, Rebecca Anastos-Wallen, Krisda Chaiyachati, Marietta Ambrose, Rupal O’Quinn, Matthew Seigerman, Lee R. Goldberg, Damien Leri, Katherine Choi, Yevginiy Gitelman, Daniel M. Kolansky, Thomas P. Cappola, Victor A. Ferrari, C. William Hanson, Mary Elizabeth Deleener, Srinath Adusumalli

**Affiliations:** 1 Division of Cardiovascular Medicine, Department of Medicine, Hospital of the University of Pennsylvania, Philadelphia; 2 Penn Cardiovascular Outcomes, Quality, and Evaluative Research Center, Cardiovascular Institute, University of Pennsylvania, Philadelphia; 3 Penn Cardiovascular Center for Health Equity and Social Justice, University of Pennsylvania, Philadelphia; 4 Leonard Davis Institute of Health Economics, University of Pennsylvania, Philadelphia; 5 Department of Biostatistics, Epidemiology, and Informatics, Perelman School of Medicine, University of Pennsylvania, Philadelphia; 6 Penn Center for Digital Cardiology, University of Pennsylvania, Philadelphia; 7 Renal-Electrolyte and Hypertension, Department of Medicine, Hospital of the University of Pennsylvania, Philadelphia; 8 Hematology and Oncology Division, Department of Medicine, Hospital of the University of Pennsylvania, Philadelphia; 9 Department of Internal Medicine, Hospital of the University of Pennsylvania, Philadelphia; 10 Penn Medicine Center for Health Care Innovation, University of Pennsylvania, Philadelphia; 11 Office of the Chief Medical Information Officer, University of Pennsylvania Health System, Philadelphia

## Abstract

**Importance:**

The coronavirus disease 2019 (COVID-19) pandemic has required a shift in health care delivery platforms, necessitating a new reliance on telemedicine.

**Objective:**

To evaluate whether inequities are present in telemedicine use and video visit use for telemedicine visits during the COVID-19 pandemic.

**Design, Setting, and Participants:**

In this cohort study, a retrospective medical record review was conducted from March 16 to May 11, 2020, of all patients scheduled for telemedicine visits in primary care and specialty ambulatory clinics at a large academic health system. Age, race/ethnicity, sex, language, median household income, and insurance type were all identified from the electronic medical record.

**Main Outcomes and Measures:**

A successfully completed telemedicine visit and video (vs telephone) visit for a telemedicine encounter. Multivariable models were used to assess the association between sociodemographic factors, including sex, race/ethnicity, socioeconomic status, and language, and the use of telemedicine visits, as well as video use specifically.

**Results:**

A total of 148 402 unique patients (86 055 women [58.0%]; mean [SD] age, 56.5 [17.7] years) had scheduled telemedicine visits during the study period; 80 780 patients (54.4%) completed visits. Of 78 539 patients with completed visits in which visit modality was specified, 35 824 (45.6%) were conducted via video, whereas 24 025 (56.9%) had a telephone visit. In multivariable models, older age (adjusted odds ratio [aOR], 0.85 [95% CI, 0.83-0.88] for those aged 55-64 years; aOR, 0.75 [95% CI, 0.72-0.78] for those aged 65-74 years; aOR, 0.67 [95% CI, 0.64-0.70] for those aged ≥75 years), Asian race (aOR, 0.69 [95% CI, 0.66-0.73]), non-English language as the patient’s preferred language (aOR, 0.84 [95% CI, 0.78-0.90]), and Medicaid insurance (aOR, 0.93 [95% CI, 0.89-0.97]) were independently associated with fewer completed telemedicine visits. Older age (aOR, 0.79 [95% CI, 0.76-0.82] for those aged 55-64 years; aOR, 0.78 [95% CI, 0.74-0.83] for those aged 65-74 years; aOR, 0.49 [95% CI, 0.46-0.53] for those aged ≥75 years), female sex (aOR, 0.92 [95% CI, 0.90-0.95]), Black race (aOR, 0.65 [95% CI, 0.62-0.68]), Latinx ethnicity (aOR, 0.90 [95% CI, 0.83-0.97]), and lower household income (aOR, 0.57 [95% CI, 0.54-0.60] for income <$50 000; aOR, 0.89 [95% CI, 0.85-0.92], for $50 000-$100 000) were associated with less video use for telemedicine visits. These results were similar across medical specialties.

**Conclusions and Relevance:**

In this cohort study of patients scheduled for primary care and medical specialty ambulatory telemedicine visits at a large academic health system during the early phase of the COVID-19 pandemic, older patients, Asian patients, and non–English-speaking patients had lower rates of telemedicine use, while older patients, female patients, Black, Latinx, and poorer patients had less video use. Inequities in accessing telemedicine care are present, which warrant further attention.

## Key Points

**Question:**

What sociodemographic factors are associated with higher use of telemedicine and the use of video (vs telephone) for telemedicine visits for ambulatory care during the coronavirus disease 2019 (COVID-19) pandemic?

**Findings:**

In this cohort study of 148 402 patients scheduled for primary care and medical specialty ambulatory telemedicine visits at a large academic health system during the early phase of the COVID-19 pandemic, older age, Asian race, non-English language as the patient’s preferred language, and Medicaid were independently associated with fewer completed telemedicine visits. Older age, female sex, Black race, Latinx ethnicity, and lower household income were associated with lower use of video for telemedicine care.

**Meaning:**

This study identified racial/ethnic, sex, age, language, and socioeconomic differences in accessing telemedicine for primary care and specialty ambulatory care; if not addressed, these differences may compound existing inequities in care among vulnerable populations.

## Teaser

This cohort study evaluates whether inequities are present in telemedicine use and video visit use for telemedicine visits during the coronavirus disease 2019 pandemic.

## Introduction

The coronavirus disease 2019 (COVID-19) pandemic has uprooted conventional health care delivery for routine ambulatory care, requiring health systems to rapidly adopt telemedicine capabilities. In response, the Centers for Medicare & Medicaid Services expanded reimbursement for ambulatory visits via telemedicine interactive communications systems, yet full reimbursement was initially restricted to visits using video, as opposed to telephone only.^1^ Although more recent regulations have improved reimbursements for telephone-only visits, there is still a lack of complete payment parity across payers for video visits.^2^ Given this, during the initial stage of the COVID-19 pandemic, many ambulatory clinicians transitioned to near-exclusive use of telemedicine, with a preference for video visits.

The use of technology to maintain access to outpatient care raises important equity concerns. The digital divide has been well documented, with lower rates of technology and broadband adoption among older patients, racial/ethnic minority groups, and those of lower socioeconomic status.^3,4,5,6,7^ A cohort study of 2940 patients scheduled at general and subspecialty cardiology clinics at our institution from March to April 2020 demonstrated that lower-income, non–English-speaking, and older patients have increased barriers to engaging in care via telemedicine,^8^ which suggests that its rapid adoption may exacerbate existing inequities.^9^

Given this, we sought to further investigate for the presence of inequities in telemedicine use more broadly across our health system. The aim of this study was to compare the demographic characteristics of patients who completed a telemedicine encounter (either telephone or video) for primary and medical specialty ambulatory care at a large academic health system with the demographic characteristics of those who were scheduled for, but did not complete, a telemedicine visit during the COVID-19 pandemic. We also identified factors associated with a completed telemedicine visit and with video use (vs telephone use) to identify inequities in telemedicine use, as well as video use specifically.

## Methods

Using the electronic medical record, we extracted demographic information for adult patients (aged ≥18 years) scheduled at our health system’s primary care and medical specialty clinics for telemedicine care from March 16 to May 11, 2020. Race and ethnicity were self-identified. The chosen study period began after implementation of a local shelter-in-place order and transition of health system ambulatory clinics to a telemedicine platform, and it ended before significant reopening of our clinics to in-person visits. During this period, direct contact was made with patients to schedule visits. Patients received appointment reminder calls and instructions for setting up video for the telemedicine visit. Medical specialties included cardiology, pulmonology, rheumatology, gastroenterology, infectious diseases, rheumatology, nephrology, hematology-oncology, and primary care (general medicine and family medicine). All ambulatory clinics in our health system, which includes 6 major referral centers and 213 clinics, were included. The catchment area covers a large urban, suburban, and semirural area encompassing parts of Pennsylvania and New Jersey. Data on median household income were obtained from the American Community Survey^10^ and linked to the patient’s zip code. This study was reviewed and classified as exempt by the University of Pennsylvania Institutional Review Board, and a waiver and exemption of informed consent was granted because the study was classified as quality improvement. This study followed the Strengthening the Reporting of Observational Studies in Epidemiology (STROBE) reporting guideline.

The primary outcomes of interest were completion of a telemedicine visit and video use (vs telephone use) for those with a completed telemedicine visit. At our institution, the electronic medical record requires clinicians to indicate that a visit occurred via telemedicine and whether the visit was conducted via video or telephone. Completion of a telemedicine visit was defined as a completion of a telemedicine encounter during the study period based on this electronic medical record indicator (as opposed to canceled without rescheduling during the study period or a no-show visit). All visits conducted in person or conducted for procedures (n = 19 995) and all patients seen in multiple specialties (n = 33 958) during the study period were excluded. For patients with multiple visits in 1 specialty, the first visit was included (eFigure in the Supplement).

### Statistical Analysis

Differences in patient characteristics between completed and noncompleted visits and between video and telephone visits were compared using χ^2^ and *t* tests as appropriate. To assess the association of sociodemographic factors with a completed telemedicine visit and separately with video use for telemedicine care, we estimated multivariable logistic regression models with completed telemedicine visit or video use as the dependent variable and age, sex, race/ethnicity, language (English or non-English), insurance payor, zip code–linked household income, and Charlson Comorbidity Index score as independent variables.^11^ The primary analysis was pooled among all general internal medicine and specialty ambulatory clinics. We additionally performed subgroup-stratified analyses, pooling specialty clinics stratified by specialty. Adjusted odds ratios (aORs) are reported with corresponding 95% CIs. Statistical analyses were performed using SAS, version 9.4 (SAS Institute Inc). All statistical testing was 2-tailed, with *P* < .05 designated as statistically significant.

## Results

A total of 148 402 patients were scheduled during the study period and met the inclusion criteria. Of those, 80 780 (54.4%) completed a telemedicine encounter, and 67 622 (45.6%) had a canceled or no-show visit. Of 78 539 patients with a completed telemedicine encounter in which visit modality was specified, 35 824 (45.6%) had video visits, and 42 715 (54.4%) had telephone visits. For all primary care and specialty ambulatory clinics, the baseline differences between patients who completed telemedicine visits and patients who did not are summarized in Table 1, and the baseline differences between patients with video visits and patients with telephone visits are summarized in Table 2. Patients with completed telemedicine visits were more likely to be younger, to be female, to have commercial insurance, and to be English speaking, and were less likely to be Asian. Patients with video use during their encounter were more likely to be younger, to be White, and to have a higher median household income.

**Table 1.** Baseline Differences Between Patients With a Completed Telemedicine Visit vs Patients Scheduled With No Telemedicine Visit in Complete Patient Cohort

Characteristic	Patients, No. (%)		*P* value
	Telemedicine visit (n = 80 780)	No telemedicine visit (n = 67 622)	
Age, y			
<55	34 752 (43.0)	27 266 (40.3)	<.001
55-64	17 467 (21.6)	14 606 (21.6)	
65-74	16 616 (20.6)	14 528 (21.5)	
≥75	11 945 (14.8)	11 222 (16.6)	
Sex			
Female	47 280 (58.5)	38 775 (57.3)	<.001
Male	33 500 (41.5)	28 847 (42.7)	
Race/ethnicity			
White	50 108 (62.0)	43 028 (63.6)	<.001
Black	19 256 (23.8)	13 127 (19.4)	
Latinx	3671 (4.5)	2829 (4.2)	
Asian	2610 (3.2)	3326 (4.9)	
Other	2031 (2.5)	1928 (2.9)	
Unknown	3103 (3.8)	3145 (4.7)	
Missing	1 (0.001)	239 (0.4)	
English language as preferred language	78 819 (97.6)	65 280 (96.5)	<.001
Missing^a^	56 (0.1)	352 (0.5)	
Payor group			
Commercial	45 127 (55.9)	37 007 (54.7)	<.001
Medicaid	6242 (7.7)	4765 (7.0)	
Medicare	28 542 (35.3)	24 335 (36.0)	
Self-pay^a^	11 (0.01)	23 (0.03)	
Missing^a^	858 (1.1)	1492 (2.2)	
Median household income, $			
<50 000	18 886 (23.4)	14 251 (21.1)	<.001
50 000-100 000	45 183 (55.9)	37 874 (56.0)	
>100 000	16 346 (20.2)	15 168 (22.4)	
Missing^a^	365 (0.5)	329 (0.5)	
Charlson Comorbidity Index score			
0	29 001 (35.9)	28 913 (42.8)	<.001
1-2	31 654 (39.2)	24 070 (35.6)	
≥3	20 125 (24.9)	14 639 (21.6)	

^a^ Not included in χ^2^ analysis.

**Table 2.** Baseline Differences Between Patients With Video Use vs Telephone Use for Telemedicine Visit for Those With Telemedicine Visit in Complete Patient Cohort

Characteristic	Patients, No. (%)		*P* value
	Telephone (n = 42 715)	Video (n = 35 824)	
Age, y			
<55	16 178 (37.9)	17 564 (49.0)	<.001
55-64	9181 (21.5)	7767 (21.7)	
65-74	9278 (21.7)	6878 (19.2)	
≥75	8078 (18.9)	3614 (10.1)	
Sex			
Female	25 497 (59.7)	20 478 (57.2)	<.001
Male	17 218 (40.3)	15 346 (42.8)	
Race/ethnicity			
White	24 540 (57.5)	24 319 (67.9)	<.001
Black	12 518 (29.3)	6089 (17.0)	
Latinx	1908 (4.5)	1615 (4.5)	
Asian	1237 (2.9)	1314 (3.7)	
Other	1043 (2.4)	933 (2.6)	
Unknown	1468 (3.4)	1554 (4.3)	
Missing^a^	1 (0.002)	0	
English language as preferred language	41 618 (97.4)	35 040 (97.8)	<.001
Missing^a^	20 (0.05)	36 (0.1)	
Payor group			
Commercial	20 696 (48.5)	23 228 (64.8)	<.001
Medicaid	3968 (9.3)	2035 (5.7)	
Medicare	17 608 (41.2)	10 169 (28.4)	
Self-pay^a^	5 (0.01)	6 (0.02)	
Missing^a^	438 (1.0)	386 (1.1)	
Median household income, $			
<50 000	12 377 (29.0)	5861 (16.4)	<.001
50 000-100 000	22 624 (53.0)	21 381 (59.7)	
>100 000	7506 (17.6)	8434 (23.5)	
Missing^a^	208 (0.5)	148 (0.4)	
Charlson Comorbidity Index score			
0	13 597 (31.8)	14 716 (41.1)	<.001
1-2	16 924 (39.6)	13 846 (38.7)	
≥3	12 194 (28.5)	7262 (20.3)	

^a^ Not included in χ^2^ analysis.

**Figure 1.**
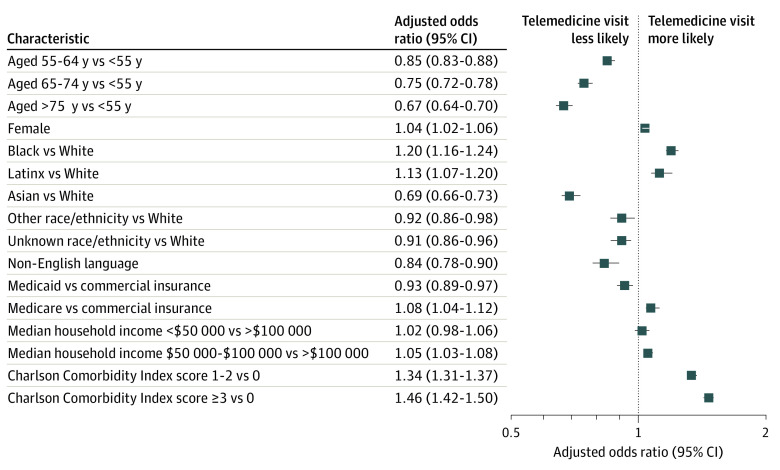
Forest Plots Showing Adjusted Odds Ratios for Telemedicine Visit Completion

Among patients scheduled at primary care and specialty ambulatory clinics, the patient characteristics associated with telemedicine visit completion on multivariable analysis are shown in Figure 1. Compared with age younger than 55 years, older age was associated with fewer completed telemedicine visits (55-64 years: aOR, 0.85 [95% CI, 0.83-0.88]; 65-74 years: aOR, 0.75 [95% CI, 0.72-0.78]; ≥75 years: aOR, 0.67 [95% CI, 0.64-0.70]). Compared with White race, Asian race was associated with fewer completed telemedicine visits (aOR, 0.69 [95% CI, 0.66-0.73]), whereas Black race (aOR, 1.20 [95% CI, 1.16-1.24]) and Latinx ethnicity (aOR, 1.13 [95% CI, 1.07-1.20]) were associated with more telemedicine visits. Female sex was associated with more telemedicine visits (aOR, 1.04 [95% CI, 1.02-1.06]). Medicaid was associated with fewer telemedicine visits compared with commercial insurance (aOR, 0.93 [95% CI, 0.89-0.97]). Non-English language as the patient’s preferred language was also independently associated with fewer completed telemedicine visits (aOR, 0.84 [95% CI, 0.78-0.90]). Compared with patients with a Charlson Comorbidity Index score of 0, patients with higher Charlson Comorbidity Index scores had higher telemedicine completion rates (Charlson Comorbidity Index score of 1-2: aOR, 1.34 [95% CI, 1.31-1.37]; Charlson Comorbidity Index score of ≥3: aOR, 1.46 [95% CI, 1.42-1.50]). Although a median household income of $50 000 to $100 000 was associated with slightly more telemedicine use compared with an income greater than $100 000 (aOR, 1.05 [95% CI, 1.03-1.08]), an income less than $50 000 was not significantly associated with telemedicine use.

**Figure 2.**
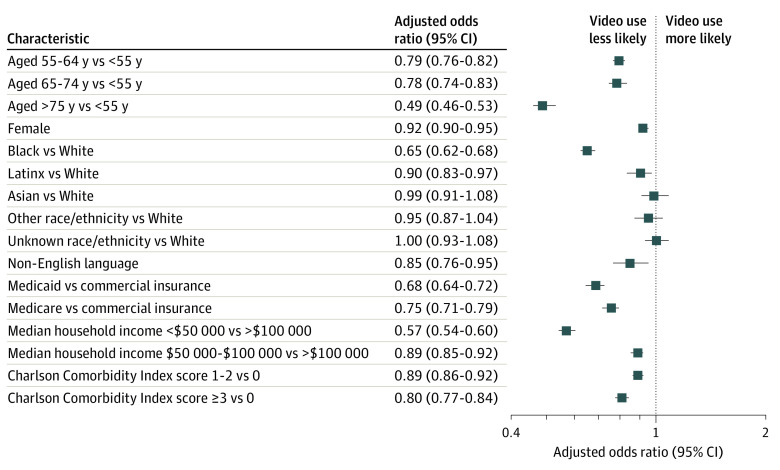
Forest Plots Showing Adjusted Odds Ratios for Video Use for Telemedicine Visit

Among patients who completed telemedicine visits in primary care and specialty ambulatory clinics during the study period, compared with patients younger than 55 years, older patients were less likely to receive care via video (55-64 years: aOR, 0.79 [95% CI, 0.76-0.82]; 65-74 years: aOR, 0.78 [95% CI, 0.74-0.83]; ≥75 years: aOR, 0.49 [95% CI, 0.46-0.53]) (Figure 2). Compared with White race, Black race (aOR, 0.65 [95% CI, 0.62-0.68]) and Latinx ethnicity (aOR, 0.90 [95% CI, 0.83-0.97]) were associated with less video use. Female sex was associated with less video use (aOR, 0.92 [95% CI, 0.90-0.95]). Compared with a median household income of more than $100 000, a lower zip code–linked household income was also associated with less video use for telemedicine care (median household income <$50 000: aOR, 0.57 [95% CI, 0.54-0.60]; median household income $50 000-$100 000: aOR, 0.89 [95% CI, 0.85-0.92]).

### Subgroup Analysis

#### Primary Care

Of 148 402 patients, 76 062 (51.3%) were included in the primary care (general medicine and family medicine) analysis. Of the 76 062 patients, 43 103 (56.7%) had a completed telemedicine visit. The baseline differences between patients who completed a telemedicine visit and scheduled patients who did not complete a telemedicine visit (canceled or no-show visit) are summarized in eTable 1 in the Supplement. Of 42 242 patients who had a telemedicine visit in primary care with visit type specified, 18 217 (43.1%) had the visit via video, whereas 24 025 (56.9%) had a telephone visit. The baseline differences between patients who had a video visit and those with a telephone visit for their telemedicine encounter in primary care are summarized in eTable 2 in the Supplement. The independent factors in multivariable analyses associated with telemedicine use and video use specifically among patients who had a telemedicine visit in primary care are summarized in eTable 3 in the Supplement.

#### Specialty Clinics and by Specialty

Of 148 402 patients, 72 340 (48.7%) were included in the specialty clinics analyses. Of 72 340 patients, 25 905 (35.8%) were included in cardiology, 14 402 (19.9%) in hematology-oncology, 9892 (13.7%) in gastroenterology, 7404 (10.2%) in endocrinology, 6608 (9.1%) in pulmonology, 4039 (5.6%) in rheumatology, 2644 (3.7%) in nephrology, and 1446 (2.0%) in infectious disease subgroup analyses.

For all specialty clinics, the baseline differences between patients who completed a telemedicine visit and those who were scheduled for a telemedicine visit but did not complete it are summarized in eTable 4 in the Supplement. Of those who completed a telemedicine visit and visit type was specified (n = 36 297), 17 607 (48.5%) had a video visit and 18 690 (51.5%) had a telephone visit. Baseline differences between patients who had a specialty clinic video visit compared with those with a specialty clinic telephone visit for their telemedicine encounter are summarized in eTable 5 in the Supplement. In multivariable analyses, the independent factors associated with telemedicine use and video use specifically among specialty clinic patients are summarized in eTable 6 in the Supplement.

Similar results were seen across specialties. The baseline differences between patients who completed a telemedicine visit and those who did not and between clinic patients who had a video visit and those with a telephone visit for their visit are summarized by specialty in eTables 7, 8, 9, 10, 11, 12, 13, and 14 in the Supplement. In multivariable analyses, the independent factors associated with completed telemedicine visits are summarized by specialty in eTable 15 in the Supplement. Older age was associated with lower telemedicine use in cardiology, pulmonology, endocrinology, rheumatology, gastroenterology, hematology-oncology, and primary care clinics. Asian race was associated with less telemedicine use in cardiology, pulmonology, nephrology, gastroenterology, rheumatology, and primary care. Non-English language as the patient’s preferred language was independently associated with fewer telemedicine visits in cardiology, nephrology, endocrinology, infectious diseases, gastroenterology, and hematology-oncology. Higher comorbidities were associated with higher telemedicine use across departments.

In multivariable analyses, the independent factors associated with video use as opposed to telephone use are summarized by specialty in eTable 16 in the Supplement. Older age was associated with less video use across all specialties. Black race, Medicaid insurance, and lower zip code–linked median income were associated with less video use in nearly all specialties.

## Discussion

The COVID-19 pandemic has devastated communities of color and marginalized populations, exposing the deep inequities of our US health care system.^12,13,14^ The findings of this study demonstrate that significant inequities are also present among patients in accessing necessary telemedicine care. These results build on initial observations on telemedicine in cardiology,^8^ revealing that inequities are pervasive throughout general medicine and specialty ambulatory care. In this study, which, to our knowledge, is the first large-scale study to characterize inequitable access to telemedical care, we found that older age, Asian race, and non-English language as the patient’s preferred language were independently associated with fewer completed telemedicine visits and that older age, Black race, Latinx ethnicity, and lower household income were associated with lower video use.

Older age was independently associated with both lower telemedicine use and lower video use. These results are consistent with evidence that older age is associated with lower internet availability, lower use of digital health technology, and slower rates of technology adoption.^15,16^ Prior studies have shown that elderly adults use online patient portals less and have privacy concerns regarding digital health use.^17^ In addition, comorbid medical conditions, along with impaired eyesight, hearing, and motor skills, make engaging in telemedical care challenging for elderly adults.^18^ However, it has been shown that, among elderly patients who are able to effectively engage in telemedicine, there are high levels of patient satisfaction and acceptance, particularly given its convenience.^18^ Although there is a lack of research on effective care delivery via telemedicine, specifically for older patients, an appropriate design of telemedicine platforms to address audio, visual, and motor impairment and the provision of broadband coverage, as well eliciting and alleviating concerns about privacy, may improve uptake in this population.^19^

Non-English language as the patient’s preferred language was independently associated with 16% lower telemedicine visit completion despite adjustment for other factors, which suggests that language barriers to care via telemedicine platforms may be prohibitive. At our institution, a more formalized outreach to contact patients in their native language, seamless end-to-end (from check-in to visit follow-up) integration of translation services into telemedicine visit technology, and translation of all visit telemedicine setup instructions into all necessary languages are being implemented to lessen this disparity.

Despite the fact that Asian individuals have high rates of technology adoption and use of broadband service,^20^ Asian race was associated with less telemedicine use. Barriers to accessing care, as well as poorer patient-doctor relationships and more frequent negative interactions with providers due to biases in care delivery have been demonstrated among some Asian American patients and may play a role in their lower rate of telemedicine use.^21,22,23^ The increase in racism against subgroups of Asian American individuals during the COVID-19 pandemic^24^ may be associated with patients’ lower rate of engagement with care, but further investigation is needed.

Video use for telemedicine visits was found to be significantly lower among Black and Latinx patients and among patients with a median household income below $50 000. These findings are likely reflective of decreased accessibility to broadband internet, connected devices, and video-capable technologies.^3,4^ Lower-income patients and patients from minority groups are less likely to own a computer, to have reliable cellphone data plans, and to have broadband internet in the home.^5^ Financial strain during the COVID-19 pandemic has already been shown to worsen the preexisting digital divide among lower-income and minority populations, many of whom struggle to pay for internet access and cellphone data plans.^7^ Provision of reliable home internet services in vulnerable zip code areas^25^ or provision of smartphones or devices equipped with data plans among these patient populations should be considered. Black and Latinx patients are overrepresented in low-paying essential industries^26^; thus, the need to continue work may preclude video use during working hours. Mistrust of telemedicine technology among Black and Latinx patients may also be a factor in the lower rates of telemedicine use among these groups.^27^ However, Black patients in our study had more completed telemedicine visits for specialty clinics, and Black and Latinx patients had higher completed telemedicine use overall and for primary care visits. Telemedicine has the potential to be leveraged to increase access to care among patient groups that may have traditionally faced barriers to in-person care. However, we must be intentional with implementation to ensure that all patients are equipped to effectively participate in telemedicine care. For example, health systems, such as ours, are initiating programs that allow “prescription” of a “connected care kit,” which include home diagnostic devices (eg, blood pressure cuff and glucometer) as well as a broadband-capable device paired with broadband service.

Female sex, while associated with increased telemedicine use overall and in primary care, was associated with less telemedicine use in specialty care and with less video use overall. Because schools have closed during the COVID-19 pandemic, it is likely that women bear a disproportionate burden of childcare duties, which may limit their time to engage in specialty telemedicine care.^28^ Women have had a greater increase in unemployment during the pandemic.^29^ Athough women may be more available for routine primary care visits, financial strains may create barriers to specialty care given the additional associated copayments.

Although there is insufficient evidence that video visits provide superior care delivery and subsequent outcomes, video visits provide several potential advantages compared with telephone visits. These include the clinician’s ability to see a patient’s home environment (and potentially conduct a home safety evaluation), to conduct a visual physical examination, to visually review medications with patients, to share screens with patients to share laboratory and study results, and to have the potential for more effective verbal cues when using translator services for non–English-speaking patients. Although recent Centers for Medicare & Medicaid Services regulations have improved reimbursement for telephone visits, these regulations are temporary and do not apply to all payers.^2^ It is critical that complete payment parity for all types of telemedicine visits, by all insurance payers, is guaranteed through permanent legislative action. Lower reimbursement for telephone visits may disproportionately and unjustly hurt clinics and clinicians that care for patients in minority groups and patients with lower income.

### Limitations

Our study has several limitations. First, we were unable to ascertain the exact barriers that patients face for completing a telemedicine or video visit. For each patient without a successful visit, it is unclear whether technical barriers—lack of computer access, broadband coverage, or lack of smartphone availability—or patient factors—privacy concerns or patient preference—were responsible. Qualitative research is under way to better characterize these barriers to guide appropriate implementation strategies. Although clinicians were equipped and strongly encouraged to preferentially use video visits at our institution, clinician preference and technological barriers on the part of clinicians may be associated with the findings. Some specialty clinicians may have more support, which allows for more time to successfully connect via video with patients. Our study represents only 1 large academic health system. This health system, however, represents primary care and medical specialty practices in 2 states covering urban, suburban, and rural areas. This study represented the period immediately after the transition to telemedicine. Differences may have been exacerbated during this abrupt transition period. We excluded patients who had in-person visits. Although it is possible that patients with barriers to telemedicine might have differentially requested in-person visits, our institution permitted in-person visits only for those who required procedures or visits that were thought to be urgent during this time. Telemedicine visits were scheduled during normal clinic hours in place of in-person visits. Allowing virtual visits during the evening or the weekend may decrease barriers to accessing care.

## Conclusions

The COVID-19 pandemic has required a dramatic shift in health care delivery, necessitating a new reliance on telemedicine. As we develop and refine our telemedicine practice, we must intentionally design our system to mitigate inequity. Engagement with community members from vulnerable populations to design and tailor connected health technologies is essential to ensure accessibility for all patients.^30^ Although many have anxiously awaited a return to “normal,” we must acknowledge that our previous “normal” was a US health care system and digital connectivity landscape fraught with inequity.^31^ As we build our telemedical health system, which is likely here to stay,^32^ a new “normal” must prioritize the needs of those who have been historically marginalized to ensure that health equity is achieved.^33^

## Notes

Supplement. **eTable 1.** Baseline Differences Between Patients With a Completed Telemedicine Visit vs Patients Scheduled With No Telemedicine Visit in Primary Care Clinics (n= 76,062) **eTable 2.** Baseline Differences Between Patients With Video Use vs Telephone Use for Telemedicine Visit for Those With Telemedicine Visit in Primary Care Clinics (n= 42,242) **eTable 3.** Multivariable Logistic Regression on Factors Associated With Telemedicine Use and Video Use for Primary Care Clinics **eTable 4.** Baseline Differences Between Patients With a Completed Telemedicine Visit vs Patients Scheduled With No Telemedicine Visit in Specialty Care Clinics (n= 72,340) **eTable 5.** Baseline Differences Between Patients With Video Use vs Telephone Use for Telemedicine Visit for Those With Telemedicine Visit in Specialty Clinics (n= 36,297) **eTable 6.** Multivariable Logistic Regression on Factors Associated With Telemedicine Use and Video Use for Specialty Clinics **eTable 7.** Baseline Differences Between Patients With Completed Telemedicine Visit vs Patients Scheduled With No Telemedicine Visit (n= 25,905) and Between Patients With Video Use vs Telephone Use for Telemedicine Visit for Those With Telemedicine Visit (n= 12775) in Cardiology Clinics **eTable 8.** Baseline Differences Between Patients With Completed Telemedicine Visit vs Patients Scheduled With No Telemedicine Visit (n= 6608) and Between Patients With Video Use vs Telephone Use for Telemedicine Visit for Those With Telemedicine Visit (n= 3365) in Pulmonology Clinics **eTable 9.** Baseline Differences Between Patients With Completed Telemedicine Visit vs Patients Scheduled With No Telemedicine Visit (n= 2644) and Between Patients With Video Use vs Telephone Use for Telemedicine Visit for Those With Telemedicine Visit (n= 1613) in Nephrology Clinics **eTable 10.** Baseline Differences Between Patients With Completed Telemedicine Visit vs Patients Scheduled With No Telemedicine Visit (n= 7404) and Between Patients With Video Use vs Telephone Use for Telemedicine Visit for Those With Telemedicine Visit (n= 3827) in Endocrinology Clinics **eTable 11.** Baseline Differences Between Patients With Completed Telemedicine Visit vs Patients Scheduled With No Telemedicine Visit (n= 1446) and Between Patients With Video Use vs Telephone Use for Telemedicine Visit for Those With Telemedicine Visit (n= 654) in Infectious Disease Clinics **eTable 12.** Baseline Differences Between Patients With Completed Telemedicine Visit vs Patients Scheduled With No Telemedicine Visit (7892) and Between Patients With Video Use vs Telephone Use for Telemedicine Visit for Those With Telemedicine Visit (n= 4754) in Gastroenterology Clinics **eTable 13.** Baseline Differences Between Patients With Completed Telemedicine Visit vs Patients Scheduled With No Telemedicine Visit (n= 4,039) and Between Patients With Video Use vs Telephone Use for Telemedicine Visit for Those With Telemedicine Visit (n= 2425) in Rheumatology Clinics. **eTable 14.** Baseline Differences Between Patients With Completed Telemedicine Visit vs Patients Scheduled With No Telemedicine Visit (14402) and Between Patients With Video Use vs Telephone Use for Telemedicine Visit for Those With Telemedicine Visit (n= 6884) in Hematology-Oncology Clinics **eTable 15.** Multivariable Logistic Regression on Factors Associated With Telemedicine Use by Specialty **eTable 16.** Multivariable Logistic Regression on Factors Associated With Video Use for Telemedicine Visit by Specialty **eFigure.** Flow Diagram of Included Patients in the Analyzed Cohort
