# Error in Abstract

**DOI:** 10.1001/jamanetworkopen.2021.1913

**Published:** 2021-02-19

In the Original Investigation titled “Patient Characteristics Associated With Telemedicine Access for Primary and Specialty Ambulatory Care During the COVID-19 Pandemic,”^1^ published December 29, 2020, there was an error in the Results paragraph of the Abstract. Both the number and the percentage of patients who had telephone visits are incorrect. The second sentence of that paragraph should have stated the following: “Of 78 539 patients with completed visits in which visit modality was specified, 35 824 (45.6%) were conducted via video, whereas 42 715 (54.4%) had telephone visits.” This article has been corrected.^1^
