# Supplementary material for: Patient Characteristics Associated With Telemedicine Access for Primary and Specialty Ambulatory Care During the COVID-19 Pandemic
Source: JAMA Netw Open. 2020 Dec 29;3(12):e2031640. doi: 10.1001/jamanetworkopen.2020.31640 (PMC7772717; doi:10.1001/jamanetworkopen.2020.31640)
Supplement: Supplement. — eTable 1. Baseline Differences Between Patients With a Completed Telemedicine Visit vs Patients Scheduled With No Telemedicine Visit in Primary Care Clinics (n= 76,062) eTable 2. Baseline Differences Between Patients With Video Use vs Telephone Use for Telemedicine Visit for Those With Telemedicine Visit in Primary Care Clinics (n= 42,242) eTable 3. Multivariable Logistic Regression on Factors Associated With Telemedicine Use and Video Use for Primary Care Clinics eTable 4. Baseline Differences Between Patients With a Completed Telemedicine Visit vs Patients Scheduled With No Telemedicine Visit in Specialty Care Clinics (n= 72,340) eTable 5. Baseline Differences Between Patients With Video Use vs Telephone Use for Telemedicine Visit for Those With Telemedicine Visit in Specialty Clinics (n= 36,297) eTable 6. Multivariable Logistic Regression on Factors Associated With Telemedicine Use and Video Use for Specialty Clinics eTable 7. Baseline Differences Between Patients With Completed Telemedicine Visit vs Patients Scheduled With No Telemedicine Visit (n= 25,905) and Between Patients With Video Use vs Telephone Use for Telemedicine Visit for Those With Telemedicine Visit (n= 12775) in Cardiology Clinics eTable 8. Baseline Differences Between Patients With Completed Telemedicine Visit vs Patients Scheduled With No Telemedicine Visit (n= 6608) and Between Patients With Video Use vs Telephone Use for Telemedicine Visit for Those With Telemedicine Visit (n= 3365) in Pulmonology Clinics eTable 9. Baseline Differences Between Patients With Completed Telemedicine Visit vs Patients Scheduled With No Telemedicine Visit (n= 2644) and Between Patients With Video Use vs Telephone Use for Telemedicine Visit for Those With Telemedicine Visit (n= 1613) in Nephrology Clinics eTable 10. Baseline Differences Between Patients With Completed Telemedicine Visit vs Patients Scheduled With No Telemedicine Visit (n= 7404) and Between Patients With Video Use vs Telephone Use for Telemedicine V [file jamanetwopen-e2031640-s001.pdf]

Supplementary Online Content

Eberly LA, Kallan MJ, Julien HM, et al. Patient characteristics associated with telemedicine access for primary and specialty ambulatory care during the COVID-19 pandemic. *JAMA Netw Open.* 2020;3(12):e2031640. doi:10.1001/jamanetworkopen.2020.31640

eTable 1. Baseline Differences Between Patients With a Completed Telemedicine Visit vs Patients Scheduled With No Telemedicine Visit in Primary Care Clinics (n= 76,062)
eTable 2. Baseline Differences Between Patients With Video Use vs Telephone Use for Telemedicine Visit for Those With Telemedicine Visit in Primary Care Clinics (n= 42,242)
eTable 3. Multivariable Logistic Regression on Factors Associated With Telemedicine Use and Video Use for Primary Care Clinics
eTable 4. Baseline Differences Between Patients With a Completed Telemedicine Visit vs Patients Scheduled With No Telemedicine Visit in Specialty Care Clinics (n= 72,340)
eTable 5. Baseline Differences Between Patients With Video Use vs Telephone Use for Telemedicine Visit for Those With Telemedicine Visit in Specialty Clinics (n= 36,297)
eTable 6. Multivariable Logistic Regression on Factors Associated With Telemedicine Use and Video Use for Specialty Clinics
eTable 7. Baseline Differences Between Patients With Completed Telemedicine Visit vs Patients Scheduled With No Telemedicine Visit (n= 25,905) and Between Patients With Video Use vs Telephone Use for Telemedicine Visit for Those With Telemedicine Visit (n= 12775) in Cardiology Clinics
eTable 8. Baseline Differences Between Patients With Completed Telemedicine Visit vs Patients Scheduled With No Telemedicine Visit (n= 6608) and Between Patients With Video Use vs Telephone Use for Telemedicine Visit for Those With Telemedicine Visit (n= 3365) in Pulmonology Clinics
eTable 9. Baseline Differences Between Patients With Completed Telemedicine Visit vs Patients Scheduled With No Telemedicine Visit (n= 2644) and Between Patients With Video Use vs Telephone Use for Telemedicine Visit for Those With Telemedicine Visit (n= 1613) in Nephrology Clinics
eTable 10. Baseline Differences Between Patients With Completed Telemedicine Visit vs Patients Scheduled With No Telemedicine Visit (n= 7404) and Between Patients With Video Use vs Telephone Use for Telemedicine Visit for Those With Telemedicine Visit (n= 3827) in Endocrinology Clinics
eTable 11. Baseline Differences Between Patients With Completed Telemedicine Visit vs Patients Scheduled With No Telemedicine Visit (n= 1446) and Between Patients With Video Use vs Telephone Use for Telemedicine Visit for Those With Telemedicine Visit (n= 654) in Infectious Disease Clinics
eTable 12. Baseline Differences Between Patients With Completed Telemedicine Visit vs Patients Scheduled With No Telemedicine Visit (7892) and Between Patients With Video Use vs Telephone Use for Telemedicine Visit for Those With Telemedicine Visit (n= 4754) in Gastroenterology Clinics
eTable 13. Baseline Differences Between Patients With Completed Telemedicine Visit vs Patients Scheduled With No Telemedicine Visit (n= 4,039) and Between Patients With Video Use vs Telephone Use for Telemedicine Visit for Those With Telemedicine Visit (n= 2425) in Rheumatology Clinics.
eTable 14. Baseline Differences Between Patients With Completed Telemedicine Visit vs Patients Scheduled With No Telemedicine Visit (14402) and Between Patients With Video Use vs Telephone Use for Telemedicine Visit for Those With Telemedicine Visit (n= 6884) in Hematology-Oncology Clinics
eTable 15. Multivariable Logistic Regression on Factors Associated With Telemedicine Use by Specialty
eTable 16. Multivariable Logistic Regression on Factors Associated With Video Use for Telemedicine Visit by Specialty
eFigure. Flow Diagram of Included Patients in the Analyzed Cohort

This supplementary material has been provided by the authors to give readers additional information about their work.

© 2020 Eberly LA et al. *JAMA Network Open.*

eTable 1. Baseline Differences Between Patients with a Completed Telemedicine Visit vs Patients Scheduled with No Telemedicine Visit in Primary Care Clinics (n= 76,062)

	**Telemedicine Visit n=43,103**	**No Telemedicine Visit n=32,959**	**p-value**
**Age, years, n (%)**			<0.001
< 55	21448 (49.8%)	15601 (47.3%)	
55-64	8937 (20.7%)	6938 (21.1%)	
65-74	7398 (17.2%)	6011 (18.2%)	
≥75	5320 (12.3%)	4409 (13.4%)	
**Gender, n (%)**			<0.001
Female	26856 (62.3%)	18888 (57.3%)	
Male	16247 (37.7%)	14071 (42.7%)	
**Race/Ethnicity, n (%)**			<0.001
White	24907 (57.8%)	19815 (60.1%)	
Black	11991 (27.8%)	7299 (22.1%)	
Latinx	2290 (5.3%)	1460 (4.4%)	
Asian	1513 (3.5%)	1931 (5.9%)	
Other	1047 (2.4%)	919 (2.8%)	
Unknown	1354 (3.1%)	1408 (4.3%)	
Missing	*1 (0.0%)*	*127 (0.4%)*	
**English Language**	41898 (97.2%)	31867 (96.7%)	0.79
Missing	32 (0.1%)	189 (0.6%)	
**Payor Group, n (%)**			<0.001
Commercial	26679 (61.9%)	20296 (61.6%)	
Medicaid	3333 (7.7%)	2151 (6.5%)	
Medicare	12437 (28.9%)	9479 (28.8%)	
Self-Pay	*4 (<0.1%)*	*8 (<0.1%)*	
Missing	*650 (1.5%)*	*1025 (3.1%)*	
**Median Household Income Category, n (%)**			<0.001
<$50K	10923 (25.3%)	7425 (22.5%)	
$50-100K	22611 (52.5%)	17278 (52.4%)	
>$100K	9398 (21.8%)	8093 (24.6%)	
Missing	171 (0.4%)	163 (0.5%)	
**Charlson Comorbidities Score, n (%)**			<0.001
0	19148 (44.4%)	17252 (52.3%)	
1-2	16303 (37.8%)	10780 (32.7%)	
≥ 3	7652 (17.8%)	4927 (14.9%)	

** Italicized categories not included in chi-squared analysis.*

© 2020 Eberly LA et al. *JAMA Network Open.*

eTable 2. Baseline Differences Between Patients with Video Use vs Telephone Use for Telemedicine Visit for Those with Telemedicine Visit in Primary Care Clinics (n= 42,242)

	**Telephone n=24,025**	**Video n=18,217**	**p-value**
**Age, years, n (%)**			<0.001
< 55	10606 (44.1%)	10388 (57.0%)	
55-64	4939 (20.6%)	3810 (20.9%)	
65-74	4481 (18.7%)	2769 (15.2%)	
≥75	3999 (16.6%)	1250 (6.9%)	
**Gender, n (%)**			<0.001
Female	15197 (63.3%)	11113 (61.0%)	
Male	8828 (36.7%)	7104 (39.0%)	
**Race/Ethnicity, n (%)**			<0.001
White	12604 (52.5%)	11906 (65.4%)	
Black	8261 (34.4%)	3406 (18.7%)	
Latinx	1198 (5.0%)	1017 (5.6%)	
Asian	747 (3.1%)	740 (4.1%)	
Other	557 (2.3%)	471 (2.6%)	
Unknown	657 (2.7%)	677 (3.7%)	
Missing	*1 (<0.1%)*	*0 (0.0%)*	
**English Language, n (%)**	23400 (97.4%)	17692 (97.1%)	0.16
Missing	*12 (<0.1%)*	*20 (0.1%)*	
**Payor Class, n (%)**			<0.001
Commercial	13002 (54.1%)	13159 (72.2%)	
Medicaid	2233 (9.3%)	1000 (5.5%)	
Medicare	8446 (35.2%)	3766 (20.7%)	
Self-Pay	*2 (<0.1%)*	*2 (<0.1%)*	
Missing	*342 (1.4%)*	*290 (1.6%)*	
**Median Household Income Category, n (%)**			<0.001
<$50K	7671 (31.9%)	2946 (16.2%)	
$50-100K	11750 (48.9%)	10457 (57.4%)	
>$100K	4502 (18.7%)	4745 (26.0%)	
Missing	*102 (0.4%)*	*69 (0.4%)*	
**Charlson Comorbidities Score, n (%)**			<0.001
0	9430 (39.3%)	9374 (51.5%)	
1-2	9314 (38.8%)	6652 (36.5%)	
≥ 3	5281 (22.0%)	2191 (12.0%)	

** Italicized categories not included in chi-squared analysis.*

© 2020 Eberly LA et al. *JAMA Network Open.*

eTable 3. Multivariable Logistic Regression on Factors Associated with Telemedicine Use and Video Use for Primary Care Clinics

	Completed Telemedicine Visit			Video Visit (vs. Telephone)		
	Adj OR	95% CI	p-value	Adj OR	95% CI	p-value
**Age (<55 years as Ref)**						
55-64	0.84	0.81-0.87	<0.001	0.80	0.76-0.85	<0.001
65-74	0.70	0.66-0.74	<0.001	0.76	0.70-0.83	<0.001
≥75	0.64	0.60-0.69	<0.001	0.41	0.37-0.45	<0.001
**Female**	1.21	1.18-1.25	<0.001	0.97	0.93-1.02	0.21
**Race/Ethnicity (White as Ref)**						
Black	1.24	1.19-1.30	<0.001	0.63	0.59-0.67	<0.001
Latinx	1.21	1.12-1.31	<0.001	0.88	0.79-0.97	0.01
Asian	0.63	0.58-0.68	<0.001	0.93	0.84-1.04	0.22
Other	0.93	0.84-1.02	0.11	0.93	0.82-1.06	0.29
Unknown	0.87	0.80-0.94	<0.001	0.99	0.88-1.11	0.88
**Non-English Language**	0.98	0.88-1.08	0.64	1.05	0.91-1.22	0.48
**Payor Class (Commercial as Ref)**						
Medicaid	1.00	0.94-1.06	0.96	0.70	0.64-0.76	<0.001
Medicare	1.14	1.08-1.21	<0.001	0.73	0.67-0.79	<0.001
**Median Household Income Category (>$100K as Ref)**						
<$50K	0.95	0.90-1.00	0.05	0.50	0.46-0.54	<0.001
$50-100K	1.04	1.00-1.08	0.05	0.87	0.83-0.92	<0.001
**Charlson Comorbidities Score (0 as Ref)**						
1-2	1.38	1.33-1.43	<0.001	0.88	0.84-0.92	<0.001
≥ 3	1.51	1.44-1.58	<0.001	0.72	0.67-0.77	<0.001

© 2020 Eberly LA et al. *JAMA Network Open.*

eTable 4. Baseline Differences Between Patients with a Completed Telemedicine Visit vs Patients Scheduled with No Telemedicine Visit in Specialty Care Clinics (n= 72,340)

	**Telemedicine Visit n=37,677**	**No Telemedicine Visit n=34,663**	**P-value**
**Age, years, n (%)**			<0.001
< 55	13304 (35.3%)	11665 (33.7%)	
55-64	8530 (22.6%)	7668 (22.1%)	
65-74	9218 (24.5%)	8517 (24.6%)	
≥75	6625 (17.6%)	6813 (19.7%)	
**Gender, n (%)**			<0.001
Female	20424 (54.2%)	19887 (57.4%)	
Male	17253 (45.8%)	14776 (42.6%)	
**Race/Ethnicity, n (%)**			<0.001
White	25201 (66.9%)	23213 (67.0%)	
Black	7265 (19.3%)	5828 (16.8%)	
Latinx	1381 (3.7%)	1369 (3.9%)	
Asian	1097 (2.9%)	1395 (4.0%)	
Other	984 (2.6%)	1009 (2.9%)	
Unknown	1749 (4.6%)	1737 (5.0%)	
Missing	*0 (0.0%)*	*112 (0.3%)*	
**English Language, n (%)**	36921 (98.0%)	33,413 (96.4%)	<0.001
Missing	*24 (0.1%)*	*163 (0.5%)*	
**Payor Class, n (%)**			0.43
Commercial	18448 (49.0%)	16711 (48.2%)	
Medicaid	2909 (7.7%)	2614 (7.5%)	
Medicare	16105 (42.7%)	14856 (42.9%)	
Self-Pay	*7 (<0.1%)*	*15 (<0.1%)*	
Missing	*208 (0.6%)*	*467 (1.3%)*	
**Median Household Income, n (%)**			<0.001
<$50K	7963 (21.1%)	6826 (19.7%)	
$50-100K	22572 (59.9%)	20596 (59.4%)	
>$100K	6948 (18.4%)	7075 (20.4%)	
Missing	*194 (0.5%)*	*166 (0.5%)*	
**Charlson Comorbidities Score, n (%)**			<0.001
0	9853 (26.2%)	11661 (33.6%)	
1-2	15351 (40.7%)	13290 (38.3%)	
≥ 3	12473 (33.1%)	9712 (28.0%)	

** Italicized categories not included in chi-squared analysis.*

© 2020 Eberly LA et al. *JAMA Network Open.*

eTable 5. Baseline Differences Between Patients with Video Use vs Telephone Use for Telemedicine Visit for Those with Telemedicine Visit in Specialty Clinics (n= 36,297)

	**Telephone n=18,690**	**Video n=17,607**	**p-value**
**Age, years, n (%)**			<0.001
< 55	5572 (29.8%)	7176 (40.8%)	
55-64	4242 (22.7%)	3957 (22.5%)	
65-74	4797 (25.7%)	4110 (23.3%)	
≥75	4079 (21.8%)	2364 (13.4%)	
**Gender, n (%)**			**<0.001**
Female	10300 (55.1%)	9365 (53.2%)	
Male	8390 (44.9%)	8242 (46.8%)	
**Race/Ethnicity, n (%)**			<0.001
White	11936 (63.9%)	12413 (70.5%)	
Black	4257 (22.8%)	2683 (15.2%)	
Latinx	710 (3.8%)	598 (3.4%)	
Asian	490 (2.6%)	574 (3.3%)	
Other	486 (2.6%)	463 (2.6%)	
Unknown	811 (4.3%)	877 (5.0%)	
**English Language, n (%)**	18218 (97.5%)	17348 (98.5%)	**<0.001**
Missing	*8 (<0.1%)*	*16 (0.1%)*	
**Payor Class, n (%)**			<0.001
Commercial	7694 (41.2%)	10069 (57.2%)	
Medicaid	1735 (9.3%)	1035 (5.9%)	
Medicare	9162 (49.0%)	6403 (36.4%)	
Self-Pay	*3 (<0.1%)*	*4 (<0.1%)*	
Missing	*96 (0.5%)*	*96 (0.5%)*	
**Median Household Income Category, n (%)**			<0.001
<$50K	4706 (25.2%)	2915 (16.6%)	
$50-100K	10874 (58.2%)	10924 (62.0%)	
>$100K	3004 (16.1%)	3689 (21.0%)	
Missing	*106 (0.6%)*	*79 (0.4%)*	
**Charlson Comorbidities Score, n (%)**			<0.001
0	4167 (22.3%)	5342 (30.3%)	
1-2	7610 (40.7%)	7194 (40.9%)	
≥ 3	6913 (37.0%)	5071 (28.8%)	

* *Italicized categories not included in chi-squared analysis.*

© 2020 Eberly LA et al. *JAMA Network Open.*

eTable 6. Multivariable Logistic Regression on Factors Associated with Telemedicine Use and Video Use for Specialty Clinics

	Completed Telemedicine Visit			Video Visit (vs. Telephone Visit)		
	**Adj OR**	**95% CI**	**p-value**	**Adj OR**	**95% CI**	**p-value**
**Age (<55 years as Ref)**						
55-64	0.89	0.85-0.92	<0.001	0.74	0.70-0.79	<0.001
65-74	0.80	0.75-0.84	<0.001	0.78	0.73-0.84	<0.001
≥75	0.69	0.65-0.73	<0.001	0.56	0.51-0.61	<0.001
**Female**	0.89	0.86-0.91	<0.001	0.91	0.87-0.95	<0.001
**Race/Ethnicity (White as Ref)**						
Black	1.06	1.02-1.12	0.01	0.77	0.72-0.83	<0.001
Latinx	0.98	0.90-1.07	0.67	0.92	0.82-1.04	0.21
Asian	0.78	0.72-0.85	<0.001	1.14	1.00-1.31	0.05
Other	0.92	0.83-1.00	0.06	0.97	0.85-1.11	0.69
Unknown	0.98	0.91-1.05	0.56	0.96	0.87-1.07	0.49
**Non-English Language**	0.69	0.62-0.77	<0.001	0.63	0.53-0.75	<0.001
**Payor Group (Commercial as Ref)**						
Medicaid	0.92	0.87-0.98	0.01	0.60	0.55-0.66	<0.001
Medicare	1.07	1.02-1.12	0.007	0.73	0.68-0.78	<0.001
**Median Household Income Category ($>100K as Ref)**						
<$50K	1.13	1.07-1.19	<0.001	0.64	0.60-0.70	<0.001
$50-100K	1.10	1.06-1.15	<0.001	0.86	0.81-0.91	<0.001
**Charlson Comorbidities Score (0 as Ref)**						
1-2	1.38	1.33-1.43	<0.001	0.82	0.78-0.87	<0.001
≥ 3	1.55	1.49-1.61	<0.001	0.74	0.70-0.79	<0.001

© 2020 Eberly LA et al. *JAMA Network Open.*

eTable 7. Baseline Differences Between Patients with Completed Telemedicine Visit vs Patients Scheduled with No Telemedicine Visit (n= 25905) and Between Patients with Video Use vs Telephone Use for Telemedicine Visit for Those with Telemedicine Visit (n= 12775) in Cardiology Clinics

	**Telemedicine Visit n=13,041**	**No Telemedicine Visit n=12,864**	**p-value**	**Telephone n=5499**	**Video n=7276**	**p-value**
**Age, years, n (%)**			0.01			<0.001
< 55	2867 (22.0%)	3029 (23.5%)		818 (14.9%)	1997 (27,4%)	
55-64	2752 (21.1%)	2676 (20.8%)		1018 (18.5%)	1668 (22.9%)	
65-74	3765 (28.9%)	3556 (27.6%)		1606 (29.2%)	2080 (28.6%)	
≥75	3657 (28.0%)	3603 (28.0%)		2057 (37.4%)	1531 (21.0%)	
**Gender, n (%)**			<0.001			<0.001
Female	5855 (44.9%)	6193 (48.1%)		2619 (47.6%)	3117 (42.8%)	
Male	7186 (55.1%)	6671 (51.9%)		2880 (52.4%)	4159 (57.2%)	
**Race/Ethnicity, n (%)**			<0.001			<0.001
White	9067 (69.5%)	9007 (70.0%)		3707 (67.4%)	5201 (71.5%)	
Black	2201 (16.9%)	1905 (14.8%)		1054 (19.2%)	1091 (15.0%)	
Latinx	568 (4.4%)	554 (4.3%)		271 (4.9%)	265 (3.6%)	
Asian	199 (1.5%)	368 (2.9%)		75 (1.4%)	120 (1.6%)	
Other	355 (2.7%)	360 (2.8%)		142 (2.6%)	206 (2.8%)	
Unknown	651 (5.0%)	652 (5.1%)		250 (4.5%)	393 (5.4%)	
Missing	*0 (0.0%)*	*18 (0.1%)*		*0 (0.0%)*	*0 (0.0%)*	
**English Language, n (%)**	12771 (97.9%)	12439 (96.7%)	<0.001	5353 (97.3%)	7165 (98.5%)	<0.001
Missing	*12 (0.1%)*	*40 (0.3%)*		*4 (0.1%)*	*8 (0.1%)*	
**Payor Group, n (%)**			0.001			<0.001
Commercial	5197 (39.9%)	5293 (41.1%)		1642 (29.9%)	3456 (47.5%)	
Medicaid	582 (4.5%)	644 (5.0%)		257 (4.7%)	310 (4.3%)	
Medicare	7219 (55.4%)	6769 (52.6%)		3583 (65.2%)	3485 (47.9%)	

© 2020 Eberly LA et al. *JAMA Network Open.*

Self-Pay	*3 (<0.1%)*	*3 (<0.1%)*		*3 (0.1%)*	*0 (0.0%)*	
Missing	*40 (0.3%)*	*155 (1.2%)*		*14 (0.3%)*	*25 (0.3%)*	
**Median Household Income, n (%)**			<0.001			<0.001
<50K	2766 (21.2%)	2524 (19.6%)		1427 (26.0%)	1263 (17.4%)	
50-100K	8216 (63.0%)	7993 (62.1%)		3380 (61.5%)	4685 (64.4%)	
>100K	1985 (15.2%)	2287 (17.8%)		657 (11.9%)	1292 (17.8%)	
Missing	*74 (0.6%)*	*60 (0.5%)*		*35 (0.6%)*	*36 (0.5%)*	
**Charlson Comorbidities Score, n (%)**			<0.001			<0.001
0	3590 (27.5%)	4266 (33.2%)		1365 (24.8%)	2163 (29.7%)	
1-2	5104 (39.1%)	4897 (38.1%)		2157 (39.2%)	2851 (39.2%)	
≥3	4347 (33.3%)	3701 (28.8%)		1977 (36.0%)	2262 (31.1%)	

© 2020 Eberly LA et al. *JAMA Network Open.*

eTable 8. Baseline Differences Between Patients with Completed Telemedicine Visit vs Patients Scheduled with No Telemedicine Visit (n= 6608) and Between Patients with Video Use vs Telephone Use for Telemedicine Visit for Those with Telemedicine Visit (n= 3365) in Pulmonology Clinics

	**Telemedicine Visit n=3543**	**No Telemedicine Visit n=3065**	**p-value**	**Telephone n=1971**	**Video n=1394**	**p-value**
**Age, years, n (%)**			0.002			<0.001
< 55	1140 (32.2%)	894 (29.2%)		494 (25.1%)	593 (42.5%)	
55-64	883 (24.9%)	713 (23.3%)		511 (25.9%)	315 (22.6%)	
65-74	896 (25.3%)	858 (28.0%)		522 (26.5%)	331 (23.7%)	
≥75	624 (17.6%)	600 (19.6%)		444 (22.5%)	155 (11.1%)	
**Gender, n (%)**			0.38			0.18
Female	2022 (57.1%)	1782 (58.1%)		1144 (58.0%)	777 (55.7%)	
Male	1521 (42.9%)	1283 (41.9%)		827 (42.0%)	617 (44.3%)	
**Race/Ethnicity, n (%)**			0.004			<0.001
White	2333 (65.8%)	1949 (63.6%)		1200 (60.9%)	1034 (74.2%)	
Black	660 (18.6%)	508 (16.6%)		440 (22.3%)	169 (12.1%)	
Latinx	128 (3.6%)	120 (3.9%)		76 (3.9%)	42 (3.0%)	
Asian	102 (2.9%)	134 (4.4%)		49 (2.5%)	47 (3.4%)	
Other	98 (2.8%)	102 (3.3%)		67 (3.4%)	26 (1.9%)	
Unknown	222 (6.3%)	178 (5.8%)		139 (7.1%)	76 (5.5%)	
Missing	*0 (0.0%)*	*74 (2.4%)*		*0 (0.0%)*	*0 (0.0%)*	
**English Language, n (%)**	3469 (97.9%)	2883 (94.1%)	0.001	1918 (97.3%)	1377 (98.8%)	0.002
Missing	*1 (<0.1%)*	*80 (2.6%)*		*0 (0.0%)*	*1 (0.1%)*	
**Payor Group, n (%)**			0.009			<0.001
Commercial	1669 (47.1%)	1305 (42.6%)		760 (38.6%)	818 (58.7%)	
Medicaid	291 (8.2%)	275 (9.0%)		185 (9.4%)	81 (5.8%)	

© 2020 Eberly LA et al. *JAMA Network Open.*

Medicare	1565 (44.2%)	1412 (46.1%)		1018 (51.6%)	485 (34.8%)	
Self-Pay	*1 (<0.1%)*	*1 (<0.1%)*		*0 (0.0%)*	*1 (0.1%)*	
Missing	*17 (0.5%)*	*72 (2.3%)*		*8 (0.4%)*	*9 (0.6%)*	
**Median Household Income, n (%)**			0.04			<0.001
<50K	669 (18.9%)	632 (20.6%)		475 (24.1%)	152 (10.9%)	
50-100K	2191 (61.8%)	1803 (58.8%)		1197 (60.7%)	880 (63.1%)	
>100K	662 (18.7%)	613 (20.0%)		284 (14.4%)	357 (25.6%)	
Missing	*21 (0.6%)*	*17 (0.6%)*		*15 (0.8%)*	*5 (0.4%)*	
**Charlson Comorbidities Score, n (%)**			<0.001			<0.001
0	763 (21.5%)	933 (30.4%)		397 (20.1%)	329 (23.6%)	
1-2	1875 (52.9%)	1437 (46.9%)		992 (50.3%)	784 (56.2%)	
≥3	905 (25.5%)	694 (22.6%)		582 (29.5%)	281 (20.2%)	

© 2020 Eberly LA et al. *JAMA Network Open.*

eTable 9. Baseline Differences Between Patients with Completed Telemedicine Visit vs Patients Scheduled with No Telemedicine Visit (n= 2644) and Between Patients with Video use vs Telephone Use for Telemedicine Visit for Those with Telemedicine Visit (n= 1613) in Nephrology Clinics

	**Telemedicine Visit n=1692**	**No Telemedicine Visit n=952**	**p-value**	**Telephone n=936**	**Video n=677**	**p-value**
**Age, years, n (%)**			0.92			<0.001
< 55	680 (40.2%)	377 (39.6%)		335 (35.8%)	313 (46.2%)	
55-64	419 (24.8%)	240 (25.2%)		237 (25.3%)	161 (23.8%)	
65-74	401 (23.7%)	220 (23.1%)		239 (25.5%)	144 (21.3%)	
≥75	192 (11.3%)	115 (12.1%)		125 (13.4%)	59 (8.7%)	
**Gender, n (%)**			0.97			0.83
Female	761 (45.0%)	429 (45.1%)		424 (45.3%)	303 (44.8%)	
Male	931 (55.0%)	523 (54.9%)		512 (55.7%)	374 (55.2%)	
**Race/Ethnicity**			0.02			<0.001
White	923 (54.6%)	481 (50.5%)		473 (50.5%)	415 (61.3%)	
Black	551 (32.6%)	299 (31.4%)		348 (37.2%)	167 (24.7%)	
Latinx	51 (3.0%)	46 (4.8%)		26 (2.8%)	24 (3.5%)	
Asian	88 (5.2%)	68 (7.1%)		43 (4.6%)	43 (6.4%)	
Other	42 (2.5%)	33 (3.5%)		28 (3.0%)	12 (1.8%)	
Unknown / Patient Declined	37 (2.2%)	24 (2.5%)		18 (1.9%)	16 (2.4%)	
Missing	*0 (0.0%)*	*1 (0.1%)*		*0 (0.0%)*	*0 (0.0%)*	
**English Language, n (%)**	1650 (97.5%)	901 (94.6%)	0.003	907 (96.9%)	664 (98.1%)	0.12
Missing	*2 (0.1%)*	*9 (0.9%)*		*1 (0.1%)*	*1 (0.1%)*	
**Payor Group, n (%)**			0.73			<0.001
Commercial	808 (47.8%)	435 (45.7%)		398 (42.5%)	377 (55.7%)	
Medicaid	134 (7.9%)	80 (8.4%)		82 (8.8%)	49 (7.2%)	
Medicare	735 (43.4%)	416 (43.7%)		447 (47.8%)	248 (36.6%)	
Self-Pay	*0 (0.0%)*	*1 (0.1%)*		*0 (0.0%)*	*0 (0.0%)*	

© 2020 Eberly LA et al. *JAMA Network Open.*

Missing	*15 (0.9%)*	*20 (2.1%)*		*9 (1.0%)*	*3 (0.4%)*	
**Median Household Income, n (%)**			0.26			<0.001
<50K	492 (29.1%)	289 (30.4%)		321 (34.3%)	150 (22.2%)	
50-100K	939 (55.5%)	497 (52.2%)		481 (51.4%)	416 (61.4%)	
>100K	257 (15.2%)	161 (16.9%)		131 (14.0%)	110 (16.2%)	
Missing/Unknown	*4 (0.2%)*	*5 (0.5%)*		*3 (0.3%)*	*1 (0.1%)*	
**Charlson Comorbidity Score, n (%)**			<0.001			0.035
0	209 (12.4%)	231 (24.3%)		103 (11.0%)	97 (14.3%)	
1-2	507 (30.0%)	278 (29.2%)		272 (29.1%)	214 (31.6%)	
≥3	976 (57.7%)	443 (46.5%)		561 (59.9%)	366 (54.1%)	

© 2020 Eberly LA et al. *JAMA Network Open.*

eTable 10. Baseline Differences Between Patients with Completed Telemedicine Visit vs Patients Scheduled with No Telemedicine Visit (n= 7404) and Between Patients With Video Use vs Telephone Use for Telemedicine Visit for Those with Telemedicine Visit (n= 3827) in Endocrinology Clinics

	**Telemedicine Visit n=4022**	**No Telemedicine Visit n=3382**	**p-value**	**Telephone n=1753**	**Video n=2074**	**p-value**
**Age, years, n (%)**			0.35			<0.001
< 55	2085 (51.8%)	1689 (49.9%)		791 (45.1%)	1187 (57.2%)	
55-64	932 (23.2%)	794 (23.5%)		449 (25.6%)	444 (21.4%)	
65-74	727 (18.1%)	656 (19.4%)		343 (19.6%)	345 (16.6%)	
≥75	278 (6.9%)	243 (7.2%)		170 (9.7%)	98 (4.7%)	
**Gender, n (%)**			<0.001			<0.001
Female	2589 (64.4%)	2351 (69.5%)		1056 (60.2%)	1405 (67.7%)	
Male	1433 (35.6%)	1031 (30.5%)		697 (39.8%)	669 (32.3%)	
**Race/Ethnicity, n (%)**			<0.001			<0.001
White	2459 (61.1%)	2058 (60.9%)		998 (56.9%)	1340 (64.6%)	
Black	961 (23.9%)	725 (21.4%)		523 (29.8%)	396 (19.1%)	
Latinx	123 (3.1%)	173 (5.1%)		54 (3.1%)	63 (3.0%)	
Asian	176 (4.4%)	180 (5.3%)		54 (3.1%)	116 (5.6%)	
Other	123 (3.1%)	93 (2.7%)		50 (2.9%)	68 (3.3%)	
Unknown	180 (4.5%)	153 (4.5%)		74 (4.2%)	91 (4.4%)	
Missing	*0 (0.0%)*	*0 (0.0%)*		*0 (0.0%)*	*0 (0.0%)*	
**English Language, n (%)**	3945 (98.1%)	3259 (96.4%)	<0.001	1716 (97.9%)	2036 (98.2%)	0.62
Missing	*1 (<0.1%)*	*3 (0.1%)*		*1 (0.1%)*	*0 (0.0%)*	
**Payor Group, n (%)**			0.37			<0.001
Commercial	2514 (62.5%)	2062 (61.0%)		944 (53.9%)	1460 (70.4%)	
Medicaid	405 (10.1%)	370 (10.9%)		221 (12.6%)	162 (7.8%)	
Medicare	1067 (26.5%)	901 (26.6%)		570 (32.5%)	437 (21.1%)	

© 2020 Eberly LA et al. *JAMA Network Open.*

Self-Pay	*1 (<0.1%)*	*5 (0.1%)*		*0 (0.0%)*	*1 (<0.1%)*	
Missing	*35 (0.9%)*	*44 (1.3%)*		*18 (1.0%)*	*14 (0.7%)*	
**Median Household Income, n (%)**			0.58			<0.001
<50K	978 (24.3%)	808 (23.9%)		518 (29.5%)	409 (19.7%)	
50-100K	2226 (55.3%)	1849 (54.7%)		924 (52.7%)	1205 (58.1%)	
>100K	802 (19.9%)	706 (20.9%)		305 (17.4%)	451 (21.7%)	
Missing	*16 (0.4%)*	*19 (0.6%)*		*6 (0.3%)*	*9 (0.4%)*	
**Charlson Comorbidities Score, n (%)**			<0.001			<0.001
0	1047 (26.0%)	1242 (36.7%)		332 (18.9%)	653 (31.5%)	
1-2	1853 (46.1%)	1371 (40.5%)		823 (46.9%)	948 (45.7%)	
≥ 3	1122 (27.9%)	769 (22.7%)		598 (34.1%)	473 (22.8%)	

© 2020 Eberly LA et al. *JAMA Network Open.*

eTable 11. Baseline Differences Between Patients with Completed Telemedicine Visit vs Patients Scheduled with No Telemedicine Visit (n= 1446) and Between Patients with Video Use vs Telephone Use for Telemedicine Visit for Those with Telemedicine Visit (n= 654) in Infectious Disease Clinics

	**Telemedicine Visit n=713**	**No Telemedicine Visit n=733**	**p-value**	**Telephone n=444**	**Video n=210**	**p-value**
**Age, years, n (%)**			0.47			<0.001
< 55	414 (58.1%)	431 (58.8%)		232 (52.3%)	144 (68.6%)	
55-64	172 (24.1%)	183 (25.0%)		114 (25.7%)	45 (21.4%)	
65-74	86 (12.1%)	90 (12.3%)		67 (15.1%)	14 (6.7%)	
≥75	41 (5.8%)	29 (4.0%)		31 (7.0%)	7 (3.3%)	
**Gender, n (%)**			0.002			0.30
Female	286 (40.1%)	236 (32.2%)		188 (42.3%)	80 (38.1%)	
Male	427 (59.9%)	497 (67.8%)		256 (57.7%)	130 (61.9%)	
**Race/Ethnicity, n (%)**			0.66			0.94
White	235 (33.0%)	255 (34.8%)		145 (32.7%)	74 (35.2%)	
Black	385 (54.0%)	365 (49.8%)		243 (54.7%)	106 (50.5%)	
Latinx	33 (4.6%)	43 (5.9%)		19 (4.3%)	10 (4.8%)	
Asian	22 (3.1%)	24 (3.3%)		13 (2.9%)	8 (3.8%)	
Other	16 (2.2%)	17 (2.3%)		10 (2.3%)	5 (2.4%)	
Unknown / Patient Declined	22 (3.1%)	28 (3.8%)		14 (3.2%)	7 (3.3%)	
Missing	*0 (0.0%)*	*1 (0.1%)*		*0 (0.0%)*	*0 (0.0%)*	
**English Language, n (%)**	700 (98.2%)	706 (96.3%)	0.06	436 (98.2%)	207 (98.6%)	0.73
Missing	*0 (0.0%)*	*2 (0.3%)*		*0 (0.0%)*	*0 (0.0%)*	
**Payor Group, n (%)**			0.40			<0.001
Commercial	263 (36.9%)	286 (39.0%)		143 (32.2%)	98 (46.7%)	
Medicaid	233 (32.7%)	215 (29.3%)		152 (34.2%)	62 (29.5%)	
Medicare	190 (26.6%)	202 (27.6%)		138 (31.1%)	40 (19.0%)	
Self-Pay	*0 (0.0%)*	*1 (0.1%)*		*0 (0.0%)*	*0 (0.0%)*	

© 2020 Eberly LA et al. *JAMA Network Open.*

Missing	*27 (3.8%)*	*29 (4.0%)*		*11 (2.5%)*	*10 (4.8%)*	
**Median Household Income, n (%)**			0.18			0.46
<50K	375 (52.6%)	360 (49.1%)		233 (52.5%)	107 (51.0%)	
50-100K	284 (39.8%)	298 (40.7%)		179 (40.3%)	82 (39.0%)	
>100K	51 (7.2%)	70 (9.5%)		30 (6.8%)	20 (9.5%)	
Missing	*3 (0.4%)*	*5 (0.7%)*		*2 (0.5%)*	*1 (0.5%)*	
**Charlson Comorbidities Score, n (%)**			0.01			0.83
0	91 (12.8%)	135 (18.4%)		55 (12.4%)	28 (13.3%)	
1-2	106 (14.9%)	96 (13.1%)		71 (16.0%)	30 (14.3%)	
≥ 3	516 (72.4%)	502 (68.5%)		318 (71.6%)	152 (72.4%)	

© 2020 Eberly LA et al. *JAMA Network Open.*

eTable 12. Baseline Differences Between Patients with Completed Telemedicine Visit vs Patients Scheduled with No Telemedicine Visit (7892) and Between Patients with Video Use vs Telephone Use for Telemedicine Visit for Those with Telemedicine Visit (n= 4754) in Gastroenterology Clinics

	**Telemedicine Visit n=4929**	**No Telemedicine Visit n=4963**	**p-value**	**Telephone n=2379**	**Video n=2375**	**p-value**
**Age, years, n (%)**			0.004			<0.001
< 55	2683 (54.4%)	2582 (52.0%)		1101 (46.3%)	1471 (61.9%)	
55-64	989 (20.1%)	1114 (22.4%)		534 (22.4%)	424 (17.9%)	
65-74	913 (18.5%)	874 (17.6%)		530 (22.3%)	359 (15.1%)	
≥75	344 (7.0%)	393 (7.9%)		214 (9.0%)	121 (5.1%)	
**Gender, n (%)**						
Female	2844 (57.7%)	3007 (60.6%)	0.004	1392 (58.5%)	1364 (57.4%)	0.45
Male	2085 (42.3%)	1956 (39.4%)		987 (41.5%)	1011 (42.6%)	
**Race/Ethnicity, n (%)**						
White	3306 (67.1%)	3199 (64.5%)		1468 (61.7%)	1715 (72.2%)	
Black	891 (18.1%)	824 (16.6%)		574 (24.1%)	288 (12.1%)	
Latinx	184 (3.7%)	195 (3.9%)		81 (3.4%)	92 (3.9%)	
Asian	218 (4.4%)	315 (6.3%)		111 (4.7%)	105 (4.4%)	
Other	124 (2.5%)	163 (3.3%)		61 (2.6%)	60 (2.5%)	
Unknown	206 (4.2%)	252 (5.1%)		84 (3.5%)	115 (4.8%)	
Missing	*0 (0.0%)*	*15 (0.3%)*		*0 (0.0%)*	*0 (0.0%)*	
**English Language, n (%)**	4827 (97.9%)	4770 (96.1%)	<0.001	2312 (97.2%)	2340 (98.5%)	0.001
Missing	*4 (0.1%)*	*16 (0.3%)*		*1 (<0.1%)*	*3 (0.1%)*	
**Payor Group, n (%)**			0.002			<0.001
Commercial	3027 (61.4%)	3190 (64.3%)		1210 (50.9%)	1701 (71.6%)	
Medicaid	538 (10.9%)	474 (9.6%)		348 (14.6%)	168 (7.1%)	
Medicare	1343 (27.2%)	1244 (25.1%)		817 (34.3%)	489 (20.6%)	

© 2020 Eberly LA et al. *JAMA Network Open.*

Self-Pay	*1 (<0.1%)*	*1 (<0.1%)*		*0 (0.0%)*	*1 (<0.1%)*	
Missing	*20 (0.4%)*	*54 (1.1%)*		*4 (0.2%)*	*16 (0.7%)*	
**Median Household Income, n (%)**			0.01			<0.001
<50K	1049 (21.3%)	980 (19.7%)		656 (27.6%)	356 (15.0%)	
50-100K	2709 (55.0%)	2689 (54.2%)		1255 (52.8%)	1365 (57.5%)	
>100K	1152 (23.4%)	1279 (25.8%)		458 (19.3%)	645 (27.2%)	
Missing	*19 (0.4%)*	*15 (0.3%)*		*10 (0.4%)*	*9 (0.4%)*	
**Charlson Comorbidities Score, n (%)**			<0.001			<0.001
0	2404 (48.8%)	2676 (53.9%)		1007 (42.3%)	1309 (55.1%)	
1-2	1464 (29.7%)	1497 (30.2%)		734 (30.9%)	685 (28.8%)	
≥3	1061 (21.5%)	790 (15.9%)		638 (26.8%)	381 (16.0%)	

© 2020 Eberly LA et al. *JAMA Network Open.*

eTable 13. Baseline Differences Between Patients with Completed Telemedicine Visit vs Patients Scheduled With No Telemedicine Visit (n= 4,039) and Between Patients with Video Use vs Telephone Use for Telemedicine Visit for Those with Telemedicine Visit (n= 2425) in Rheumatology Clinics

	**Telemedicine Visit n=2558**	**No Telemedicine Visit n=1481**	**p-value**	**Telephone n=1486**	**Video n=939**	**p-value**
**Age, years, n (%)**			0.004			<0.001
< 55	1288 (50.4%)	699 (47.2%)		687 (46.2%)	530 (56.4%)	
55-64	681 (26.6%)	371 (25.1%)		418 (28.1%)	227 (24.2%)	
65-74	420 (16.4%)	275 (18.6%)		247 (16.6%)	156 (16.6%)	
≥75	169 (6.6%)	136 (9.2%)		134 (9.0%)	26 (2.8%)	
**Gender, (%)**			0.47			0.97
Female	2010 (78.6%)	1178 (79.5%)		1164 (78.3%)	735 (78.3%)	
Male	548 (21.4%)	303 (20.5%)		322 (21.7%)	204 (21.7%)	
**Race/Ethnicity, n (%)**			<0.001			<0.001
White	1567 (61.3%)	938 (63.3%)		866 (58.3%)	624 (66.5%)	
Black	581 (22.7%)	267 (18.0%)		385 (25.9%)	154 (16.4%)	
Latinx	122 (4.8%)	54 (3.6%)		78 (5.2%)	42 (4.5%)	
Asian	76 (3.0%)	66 (4.5%)		44 (3.0%)	29 (3.1%)	
Other	79 (3.1%)	52 (3.5%)		43 (2.9%)	32 (3.4%)	
Unknown	133 (5.2%)	104 (7.0%)		70 (4.7%)	58 (6.2%)	
Missing	*0 (0.0%)*	*0 (0.0%)*		*0 (0.0%)*	*0 (0.0%)*	
**English Language, n (%)**	2499 (97.7%)	1446 (97.6%)	0.99	1437 (96.7%)	931 (99.1%)	<0.001
Missing	*0 (0.0%)*	*1 (0.1%)*		*0 (0.0%)*	*0 (0.0%)*	
**Payor Group, n (%)**			0.34			<0.001
Commercial	1477 (57.7%)	860 (58.1%)		763 (51.3%)	642 (68.4%)	
Medicaid	329 (12.9%)	167 (11.3%)		220 (14.8%)	87 (9.3%)	
Medicare	724 (28.3%)	432 (29.2%)		486 (32.7%)	200 (21.3%)	

© 2020 Eberly LA et al. *JAMA Network Open.*

Self-Pay	*1 (<0.1%)*	*1 (0.1%)*		*0 (0.0%)*	*1 (0.1%)*	
Missing	*27 (1.1%)*	*21 (1.4%)*		*17 (1.1%)*	*9 (1.0%)*	
**Median Household Income, n(%)**			0.01			<0.001
<50K	648 (25.3%)	321 (21.7%)		430 (28.9%)	173 (18.4%)	
50-100K	1418 (55.4%)	837 (56.5%)		807 (54.3%)	554 (59.0%)	
>100K	480 (18.8%)	316 (21.3%)		240 (16.2%)	209 (22.3%)	
Missing/Unknown	*12 (0.5%)*	*7 (0.5%)*		*9 (0.6%)*	*3 (0.3%)*	
**Charlson Comorbidity Score, n (%)**			<0.001			<0.001
0	674 (26.3%)	606 (40.9%)		344 (23.1%)	297 (31.6%)	
1-2	1423 (55.6%)	646 (43.6%)		834 (56.1%)	511 (54.4%)	
≥ 3	461 (18.0%)	229 (15.5%)		308 (20.7%)	131 (14.0%)	

© 2020 Eberly LA et al. *JAMA Network Open.*

eTable 14. Baseline Differences Between Patients with Completed Telemedicine Clinic vs Patients Scheduled with No Telemedicine Visit (14402) and Between Patients With Video Use vs Telephone Use for Telemedicine Visit for Those with Telemedicine Visit (n= 6884) in Hematology-Oncology Clinics

	**Telemedicine Visit n=7179**	**No Telemedicine Visit n=7223**	**p-value**	**Telephone n=4222**	**Video n=2662**	**p-value**
**Age, years, n (%)**			<0.001			<0.001
< 55	2147 (29.9%)	1964 (27.2%)		1114 (26.4%)	941 (35.3%)	
55-64	1702 (23.7%)	1577 (21.8%)		961 (22.8%)	673 (25.3%)	
65-74	2010 (28.0%)	1988 (27.5%)		1243 (29.4%)	681 (25.6%)	
≥75	1320 (18.4%)	1694 (23.5%)		904 (21.4%)	367 (13.8%)	
**Gender, n (%)**			<0.001			<0.001
Female	4057 (56.5%)	4711 (65.2%)		2313 (54.8%)	1584 (59.5%)	
Male	3122 (43.5%)	2512 (34.8%)		1909 (45.2%)	1078 (40.5%)	
**Race/Ethnicity, n (%)**			0.009			<0.001
White	5311 (74.0%)	5326 (73.7%)		3079 (72.9%)	2010 (75.5%)	
Black	1035 (14.4%)	935 (12.9%)		690 (16.3%)	312 (11.7%)	
Latinx	172 (2.4%)	184 (2.5%)		105 (2.5%)	60 (2.3%)	
Asian	216 (3.0%)	240 (3.3%)		101 (2.4%)	106 (4.0%)	
Other	147 (2.0%)	189 (2.6%)		85 (2.0%)	53 (2.0%)	
Unknown	298 (4.2%)	346 (4.8%)		162 (3.8%)	121 (4.5%)	
Missing	*0 (0.0%)*	*3 (<0.1%)*		*0 (0.0%)*	*0 (0.0%)*	
**English Language, n (%)**	7060 (98.3%)	7009 (97.0%)	<0.001	4139 (98.0%)	2628 (98.7%)	0.01
Missing	*4 (0.1%)*	*12 (0.2%)*		*1 (<0.1%)*	*3 (0.1%)*	
**Payor Group, n (%)**			0.001			<0.001
Commercial	3493 (48.7%)	3280 (45.4%)		1834 (43.4%)	1517 (57.0%)	
Medicaid	397 (5.5%)	389 (5.4%)		270 (6.4%)	116 (4.4%)	
Medicare	3262 (45.4%)	3480 (48.2%)		2103 (49.8%)	1019 (38.3%)	

© 2020 Eberly LA et al. *JAMA Network Open.*

Self-Pay	*0 (0.0%)*	*2 (<0.1%)*		*0 (0.0%)*	*0 (0.0%)*	
Missing	*27 (0.4%)*	*72 (1.0%)*		*15 (0.4%)*	*10 (0.4%)*	
**Median Household Income, n (%)**			0.08			<0.001
<50K	986 (13.7%)	912 (12.6%)		646 (15.3%)	305 (11.5%)	
50-100K	4589 (63.9%)	4630 (64.1%)		2651 (62.8%)	1737 (65.3%)	
>100K	1559 (21.7%)	1643 (22.7%)		899 (21.3%)	605 (22.7%)	
Missing	*45 (0.6%)*	*38 (0.5%)*		*26 (0.6%)*	*15 (0.6%)*	
**Charlson Comorbidities Score, n (%)**			<0.001			<0.001
0	1075 (15.0%)	1572 (21.8%)		564 (13.4%)	466 (17.5%)	
1-2	3019 (42.1%)	3068 (42.5%)		1727 (40.9%)	1171 (44.0%)	
≥3	3085 (43.0%)	2583 (35.8%)		1931 (45.7%)	1025 (38.5%)	

© 2020 Eberly LA et al. *JAMA Network Open.*

eTable 15. Multivariable Logistic Regression on Factors Associated with Telemedicine Use by Specialty

	Cardiology	Pulmonology	Nephrology	Endocrinology	Infectious Disease	Gastroenterology	Rheumatology	Hematology-Oncology
	OR [95% CI]	OR [95% CI]	OR [95% CI]	OR [95% CI]	OR [95% CI]	OR [95% CI]	OR [95% CI]	OR [95% CI]
**Age (Ref <55 years)**								
55-64	1.01 [0.94-1.09]	0.92 [0.80-1.06]	0.85 [0.68-1.05]	**0.84 [0.75-0.95]**	1.04 [0.80-1.35]	**0.76 [0.68-0.84]**	0.91 [0.77-1.08]	**0.86 [0.78-0.95]**
65-74	0.97 [0.88-1.06]	**0.73 [0.61-0.87]**	0.92 [0.71-1.18]	**0.75 [0.63-0.90]**	1.16 [0.78-1.73]	**0.75 [0.64-0.87]**	**0.68 [0.53-0.86]**	**0.73 [0.65-0.83]**
≥75	**0.90 [0.81-0.99]**	**0.71 [0.58-0.87]**	0.81 [0.59-1.10]	**0.75 [0.59-0.94]**	**1.79 [1.01-3.17]**	**0.61 [0.50-0.75]**	**0.50 [0.37-0.69]**	**0.55 [0.48-0.63]**
**Female**	**0.89 [0.84-0.93]**	**0.90 [0.81-0.99]**	1.09 [0.92-1.28]	**0.86 [0.77-0.95]**	**1.44 [1.15-1.81]**	**0.89 [0.82-0.97]**	**0.84 [0.71-0.99]**	**0.69 [0.65-0.74]**
**Race/Ethnicity (White as Ref)**								
Black	**1.11 [1.03-1.20]**	1.17 [0.99-1.37]	0.91 [0.73-1.14]	1.09 [0.94-1.26]	0.91 [0.67-1.24]	0.93 [0.82-1.06]	1.13 [0.92-1.40]	1.09 [0.97-1.22]
Latinx	1.14 [1.00-1.31]	0.95 [0.71-1.25]	0.64 [0.40-1.00]	**0.62 [0.48-0.80]**	0.99 [0.58-1.71]	0.91 [0.73-1.13]	1.18 [0.82-1.69]	0.99 [0.79-1.25]
Asian	**0.61 [0.51-0.74]**	**0.72 [0.54-0.97]**	**0.68 [0.47-0.98]**	0.89 [0.71-1.12]	1.22 [0.63-2.37]	**0.74 [0.61-0.90]**	**0.63 [0.44-0.91]**	1.00 [0.81-1.22]
Other	1.00 [0.86-1.17]	0.83 [0.62-1.11]	0.70 [0.43-1.15]	1.12 [0.84-1.50]	1.13 [0.54-2.38]	**0.77 [0.61-0.98]**	0.93 [0.63-1.35]	0.80 [0.64-1.00]
Unknown	1.02 [0.91-1.14]	1.11 [0.90-1.38]	0.93 [0.53-1.61]	1.07 [0.85-1.34]	1.09 [0.58-2.05]	0.85 [0.70-1.03]	0.80 [0.61-1.06]	0.96 [0.81-1.13]
**Non-English Language**	**0.68 [0.57-0.82]**	0.80 [0.56-1.13]	0.73 [0.44-1.20]	**0.61 [0.44-0.84]**	**0.45 [0.21-0.96]**	**0.61 [0.46-0.80]**	1.29 [0.78-2.11]	**0.60 [0.47-0.78]**
**Payor Class (Commercial as Ref)**								
Medicaid	**0.82 [0.72-0.93]**	**0.77 [0.63-0.93]**	0.94 [0.68-1.29]	0.89 [0.75-1.05]	1.13 [0.86-1.49]	**1.20 [1.03-1.38]**	0.99 [0.79-1.24]	0.94 [0.80-1.11]
Medicare	1.07 [0.99-1.16]	0.96 [0.83-1.13]	0.85 [0.69-1.06]	1.02 [0.86-1.19]	0.85 [0.62-1.17]	**1.27 [1.10-1.46]**	1.13 [0.91-1.41]	1.08 [0.97-1.20]
**Median Household Income (>100K as Ref)**								
<50K	**1.22 [1.12-1.34]**	0.94 [0.78-1.13]	1.01 [0.75-1.35]	1.00 [0.85-1.19]	1.50 [0.96-2.36]	1.11 [0.97-1.28]	1.10 [0.87-1.40]	1.08 [0.95-1.24]
50-100K	**1.17 [1.09-1.25]**	**1.16 [1.02-1.32]**	1.14 [0.90-1.44]	1.05 [0.93-1.19]	1.44 [0.95-2.17]	1.07 [0.97-1.18]	1.05 [0.88-1.24]	1.04 [0.96-1.13]
**Charlson Comorbidity Score (0 as Ref)**								
1-2	**1.21 [1.14-1.29]**	**1.60 [1.41-1.81]**	**2.02 [1.58-2.59]**	**1.58 [1.41-1.76]**	**1.55 [1.04-2.32]**	**1.11 [1.01-1.22]**	**2.01 [1.73-2.34]**	**1.47 [1.34-1.62]**
≥3	**1.34 [1.25-1.43]**	**1.66 [1.43-1.93]**	**2.64 [2.07-3.37]**	**1.77 [1.55-2.03]**	**1.53 [1.11-2.11]**	**1.52 [1.35-1.71]**	**1.91 [1.55-2.35]**	**1.81 [1.64-2.00]**

Bolded confidence intervals represent significant p values at <0.05

© 2020 Eberly LA et al. *JAMA Network Open.*

eTable 16. Multivariable Logistic Regression on Factors Associated with Video Use for Telemedicine Visit by Specialty

	Cardiology	Pulmonology	Nephrology	Endocrinology	Infectious Disease	Gastroenterology	Rheumatology	Hematology-Oncology
	OR [95% CI]	OR [95% CI]	OR [95% CI]	OR [95% CI]	OR [95% CI]	OR [95% CI]	OR [95% CI]	OR [95% CI]
**Age (Ref <55 years)**								
55-64	**0.65 [0.58-0.73]**	**0.53 [0.43-0.64]**	**0.75 [0.58-0.98]**	**0.75 [0.63-0.89]**	**0.57 [0.37-0.88]**	**0.67 [0.57-0.78]**	**0.74 [0.60-0.91]**	**0.86 [0.75-0.98]**
65-74	**0.57 [0.49-0.65]**	**0.62 [0.48-0.80]**	0.75 [0.55-1.03]	0.98 [0.76-1.26]	**0.33 [0.16-0.69]**	**0.70 [0.56-0.88]**	1.07 [0.79-1.44]	**0.76 [0.63-0.91]**
≥75	**0.34 [0.29-0.39]**	**0.35 [0.26-0.47]**	**0.65 [0.43-0.97]**	**0.60 [0.43-0.85]**	**0.33 [0.12-0.88]**	**0.58 [0.43-0.79]**	**0.34 [0.21-0.57]**	**0.59 [0.48-0.72]**
**Female**	**0.88 [0.81-0.94]**	0.98 [0.85-1.14]	1.00 [0.81-1.24]	**1.28 [1.11-1.48]**	0.90 [0.63-1.29]	0.96 [0.85-1.08]	1.13 [0.91-1.39]	**1.16 [1.04-1.28]**
**Race/Ethnicity (White as Ref)**								
Black	**0.86 [0.77-0.96]**	**0.65 [0.51-0.83]**	**0.68 [0.51-0.90]**	**0.74 [0.60-0.91]**	0.74 [0.45-1.21]	**0.66 [0.55-0.80]**	0.78 [0.59-1.01]	**0.75 [0.63-0.89]**
Latinx	**0.73 [0.60-0.90]**	0.70 [0.45-1.07]	1.37 [0.73-2.55]	0.92 [0.62-1.37]	0.81 [0.34-1.96]	1.17 [0.84-1.63]	1.17 [0.76-1.82]	0.90 [0.64-1.26]
Asian	0.99 [0.72-1.34]	1.10 [0.69-1.75]	1.30 [0.80-2.12]	**1.67 [1.17-2.39]**	1.18 [0.42-3.28]	0.87 [0.65-1.18]	1.04 [0.62-1.76]	**1.79 [1.31-2.43]**
Other	1.08 [0.86-1.36]	**0.47 [0.29-0.75]**	0.54 [0.27-1.10]	1.11 [0.75-1.64]	0.91 [0.29-2.91]	0.98 [0.67-1.44]	1.27 [0.77-2.09]	0.98 [0.68-1.40]
Unknown	1.01 [0.85-1.20]	**0.58 [0.42-0.78]**	1.05 [0.52-2.15]	0.83 [0.60-1.15]	0.89 [0.33-2.40]	1.06 [0.78-1.43]	1.08 [0.74-1.59]	1.11 [0.86-1.42]
**Non-English Language**	0.80 [0.60-1.07]	0.56 [0.29-1.05]	0.51 [0.24-1.09]	0.92 [0.55-1.54]	0.81 [0.18-3.56]	**0.56 [0.35-0.90]**	**0.27 [0.12-0.59]**	**0.51 [0.32-0.82]**
**Payor Class (Commercial as Ref)**								
Medicaid	**0.72 [0.60-0.87]**	**0.65 [0.48-0.88]**	0.89 [0.59-1.34]	**0.61 [0.48-0.78]**	**0.61 [0.40-0.95]**	**0.50 [0.40-0.62]**	**0.63 [0.47-0.85]**	**0.59 [0.46-0.75]**
Medicare	**0.76 [0.68-0.85]**	**0.67 [0.53-0.84]**	**0.75 [0.57-0.98]**	**0.62 [0.49-0.78]**	0.71 [0.42-1.21]	**0.64 [0.52-0.79]**	**0.63 [0.48-0.83]**	**0.81 [0.69-0.94]**
**Median Household Income (>100K as Ref)**								
<50K	**0.49 [0.42-0.56]**	**0.34 [0.25-0.45]**	0.76 [0.52-1.10]	**0.71 [0.55-0.90]**	0.59 [0.29-1.23]	**0.56 [0.46-0.69]**	**0.60 [0.44-0.82]**	0.89 [0.72-1.08]
50-100K	**0.72 [0.65-0.81]**	**0.60 [0.50-0.72]**	1.13 [0.85-1.52]	0.89 [0.75-1.07]	0.52 [0.26-1.02]	**0.83 [0.72-0.96]**	**0.77 [0.61-0.96]**	1.00 [0.88-1.13]
**Charlson Comorbidity Score (0 as Ref)**								
1-2	0.99 [0.90-1.09]	1.12 [0.93-1.36]	0.90 [0.64-1.27]	**0.67 [0.56-0.79]**	0.91 [0.47-1.75]	**0.86 [0.75-0.99]**	**0.80 [0.66-0.98]**	**0.86 [0.74-0.99]**
≥3	1.02 [0.92-1.12]	0.91 [0.73-1.15]	0.96 [0.68-1.34]	**0.59 [0.49-0.72]**	1.00 [0.57-1.75]	**0.67 [0.56-0.79]**	**0.67 [0.51-0.88]**	**0.75 [0.64-0.88]**

Bolded confidence intervals represent significant p values at <0.05.

© 2020 Eberly LA et al. *JAMA Network Open.*

eFigure. Flow Diagram of Included Patients in the Analyzed Cohort

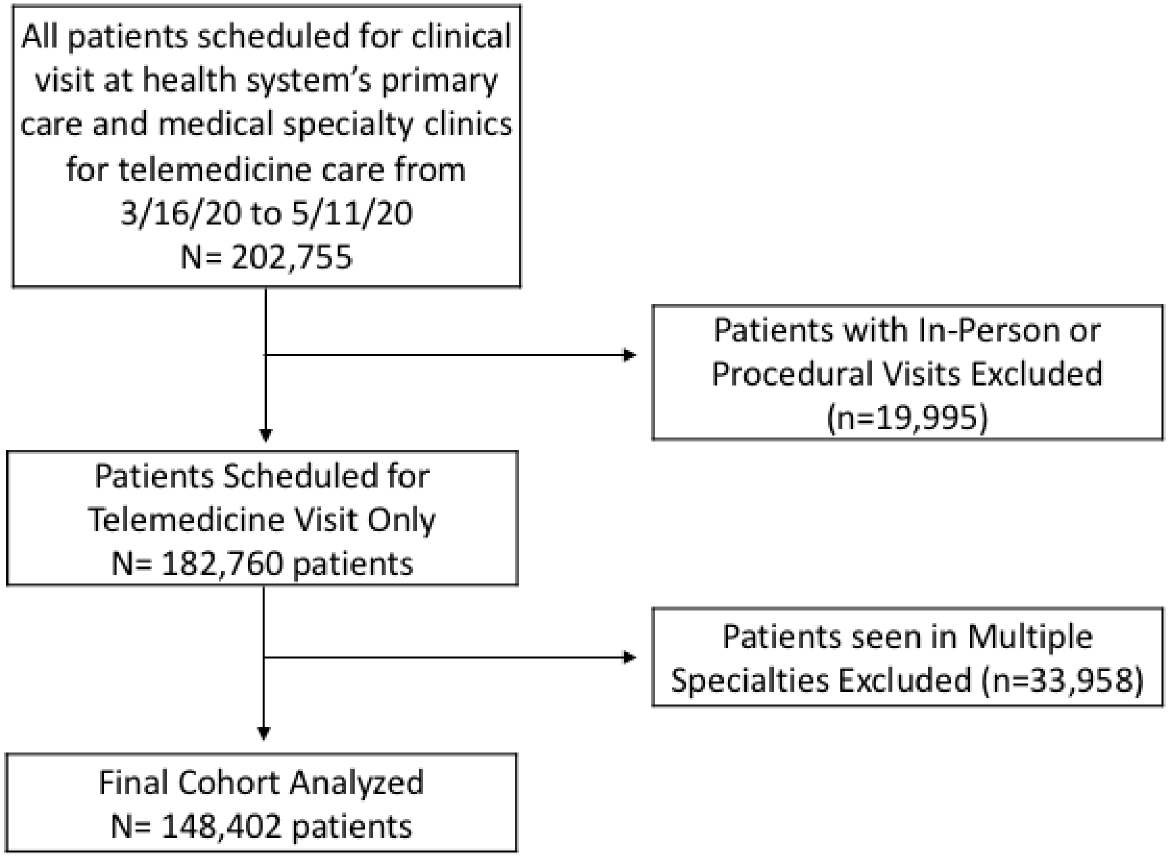

© 2020 Eberly LA et al. *JAMA Network Open.*
